# Maritoclax Overcomes FBW7 Deficiency‐Driven Irinotecan Resistance in Colorectal Cancer by Targeting MCL1

**DOI:** 10.1002/cam4.71419

**Published:** 2025-11-28

**Authors:** Qian Lin, Shuting Liu, Hongfei Jiang, Shasha Wang, Yixin Duan

**Affiliations:** ^1^ Key Laboratory of Marine Drugs, Chinese Ministry of Education, School of Medicine and Pharmacy Ocean University of China Qingdao China; ^2^ Qingdao Cancer Institute, The Affiliated Hospital of Qingdao University, Qingdao University Qingdao China; ^3^ Department of Oncology The Affiliated Hospital of Qingdao University Qingdao China; ^4^ College of Food Science and Engineering Ocean University of China Qingdao China

**Keywords:** colorectal cancer, FBW7, irinotecan, Maritoclax, MCL1

## Abstract

**Introduction:**

FBW7, a tumor suppressive E3 ubiquitin ligase frequently mutated in colorectal cancer (CRC), mediates chemotherapy resistance. While irinotecan (via its active metabolite SN38) is a first‐line TOP1 inhibitor for advanced CRC, the mechanistic link between FBW7 dysfunction and irinotecan resistance remains elusive.

**Methods:**

CRISPR/Cas9 gene editing and RNA interference were applied to establish FBW7‐knockout and ‐knockdown CRC cells. CCK8 assay was performed to detect the inhibition ratio of SN38 and Maritoclax on CRC cells. Western blot, immunochemistry, RT‐qPCR and co‐immunoprecipitation were performed to detect the expression and interaction of FBW7 and MCL1. The conformational change of the FBW7 R465C mutation and its interaction with substrates were elucidated through AlphaFold. An animal experiment was performed to detect the in vivo therapeutic effect of irinotecan and Maritoclax.

**Results:**

FBW7 deficiency (including loss, low expression and mutation) reduced SN38 sensitivity and upregulated MCL1 expression in both CRC cells and patient tissues. The R465C mutation disrupted FBW7‐MCL1 binding and stabilized MCL1. Moreover, SN38 modulated MCL1 expression, elevated MCL1 expression led to SN38 resistance in CRC cells. Maritoclax, a specific MCL1 inhibitor, reversed irinotecan/SN38 resistance in FBW7‐deficient CRC cells and xenograft models, synergizing with irinotecan to suppress tumor growth.

**Conclusions:**

FBW7 deficiency induces irinotecan resistance via MCL1 stabilization, which is therapeutically exploitable by Maritoclax. Our work identifies MCL1 inhibition as a precision strategy for FBW7‐deficient CRC and supports clinical translation of Maritoclax‐irinotecan combinations.

## Graphical abstract

FBW7 deficiency (including loss, low expression and mutation) leads to upregulation of MCL1 protein, resulting in irinotecan/SN38 resistance in CRC; inhibition of MCL1 by Maritoclax enhanced the antitumor efficacy of irinotecan/SN38.

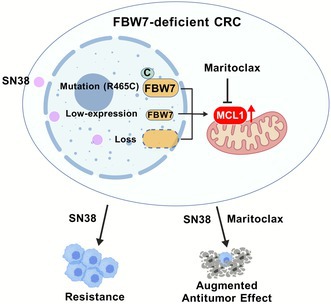

## 1 Introduction

Colorectal cancer (CRC) ranks as the third most common cancer globally (9.6% of cases) and the second leading cause of cancer deaths (9.3%) [1]. More than 50% of CRC patients in the United States are diagnosed at an advanced stage (Stage III or later) [2], with patients having metastatic forms of the disease exhibiting a dismal 5‐year survival rate of ~10% [2]. For early‐stage CRC, surgical resection remains the primary curative intervention. Advanced CRC relies heavily on systemic chemotherapy; the FOLFIRI regimen (leucovorin, fluorouracil, and irinotecan) is routinely adopted as the standard first‐line systemic chemotherapy treatment. However, chemotherapy resistance remains a major barrier to improving outcomes [3].

Irinotecan, a cornerstone of FOLFIRI regimen, exerts its cytotoxicity via its active metabolite SN38, which stabilizes TOP1‐DNA cleavage complexes, causing lethal DNA damage during replication [4, 5, 6]. Despite its efficacy, intrinsic and acquired resistance to irinotecan/SN38 is common and mechanistically heterogeneous [7], involving: TOP1 gene mutations [8], tumor cell stress responses to SN38‐TOP1‐DNA ternary complex [9, 10], activation of pro‐survival signaling pathways in cancer cells [11, 12] and epigenetic changes [13], etc.

The tumor suppressor FBW7, an E3 ubiquitin ligase, plays a critical role in regulating cell division, proliferation, and differentiation [14]. In CRC, approximately 16% of cases harbor FBW7 mutations, which promote tumorigenesis and progression [15, 16, 17]. R465, R479 and R505 of FBW7 have been identified as three hallmark cancer hotspot arginine residues. The R465C mutation in the WD40 domain of FBW7 is the most frequently observed pathogenic site [15] and is linked to poor survival and therapy resistance [18, 19]. FBW7 targets key proteins (e.g., Cyclin E, c‐Myc, MCL1) for degradation; its dysfunction leads to their aberrant accumulation in various biological processes [16]. Although FBW7 deficiency is known to confer resistance to chemotherapeutic agents [20, 21, 22, 23, 24, 25, 26, 27], its role in irinotecan resistance remains unclear.

FBW7 deficiency stabilizes MCL1, an anti‐apoptotic Bcl‐2 family protein that promotes cell survival [28]. Elevated MCL1 is a hallmark of FBW7‐deficient CRC and drives resistance to regorafenib [20, 27], sorafenib [27], microtubule‐targeting agents [25], and Hsp90 inhibitors [26]. Critically, inhibition of MCL1 protein has been shown to resensitize tumors to these chemotherapeutic agents.

Marinopyrrole A (Maritoclax) is a specific MCL1 inhibitor; it is a natural product derived from a marine actinomycete strain CNQ‐418. Studies have demonstrated that Maritoclax directly binds to MCL1 and promotes its degradation to induce apoptosis in cancer cells [29, 30, 31, 32, 33]. It also enhances the pro‐apoptotic effects of Bcl‐2 family inhibitors, such as ABT‐737 [29, 31] and ABT‐263 (Navitoclax) [33]. However, its potential to reverse irinotecan resistance in FBW7‐deficient CRC is unexplored.

Here, we demonstrate that FBW7 deficiency (including loss, low expression, and mutation) upregulates MCL1, driving irinotecan/SN38 resistance in CRC. Strikingly, the MCL1 inhibitor Maritoclax overcomes this resistance, restoring irinotecan sensitivity in FBW7‐deficient models in vitro and in vivo. Our work identifies Maritoclax as a precision therapy for FBW7‐deficient CRC and provides a mechanistic basis for clinical translation.

## 2 Materials and Methods

### 2.1 Cell Culture and Transfection

Human colorectal cancer cell lines HCT‐116, Lim1215, Lim2405, DLD1, RKO, SW1463, SW48, HCT‐8 and LOVO were obtained from ATCC. Genotypes of top CRC‐related genes in these cell lines were checked at Cellosaurus (https://www.cellosaurus.org/index.html) listed in Table S1. Cells were cultured at 37°C and 5% CO2 in a McCoy's 5A modified media (Cat# 12330031; Gibico) or DMEM (high glucose, Cat# C1199500BT; Gibico) with 10% FBS (Cat# AC03L054; Heyuan Liji), 100 U/mL penicillin and 100 mg/mL streptomycin (Cat# P1400; Solarbio). All cell lines were routinely screened for the presence of mycoplasma by Mycoplasma PCR Detection Kit (Cat# C0301S; Beyotime Biotechnology). Cells were seeded in 6/96 well plates at 50%–60% confluency before drug treatment and in 6/12 well plates or a 10 cm dish at 80% confluency for transfection. Opti‐MEM (Cat# 31985070; Invitrogen), Lipofectamine 2000 (Cat# 11668019; Life Technologies) and Polyethylenimine (PEI, Cat# 23966‐2; Polysciences) were used for transfection.

### 2.2 Plasmids, Antibodies and Reagents

Human FBW7 and MCL1 cDNAs were obtained from RKO cells and cloned into pcDNA3.1‐N‐Flag, pcDNA3.1‐N‐HA or pLVX‐C‐HA (Cat# VT9011; Youbio) expression vector. LentiCRISPRv2 plasmid was obtained from Addgene (Cat# 98290). The gRNA sequence of the FBW7 gene is 5′‐ccattccacttgttaacgac‐3′ and the sgCtrl sequence is 5′‐cttccgcggcccgttcaa‐3′ (Addgene plasmid #107402). The reagents used for molecular cloning were KOD‐Plus‐Neo (Cat# KOD‐401; Toyobo), E.Z.N.A. Gel Extraction Kit (Cat# D2500; Omega Bio‐TEK), Fastdigest restriction enzymes (Thermofisher), BsmBI‐v2 (Cat# R0580; NEB), T4 DNA ligase (Cat# EL0011; Thermoscientific) and XL10‐Gold Chemically Competent Cell (Cat# C1450; Solarbio). FBW7 shRNAs (Cat# sc‐37547‐SH) were purchased from Santa Cruz Biotechnology. Plasmids were purified by the EndoFree Mini Plasmid Kit II (Cat# 4992422; TIANGEN). Sequencing was performed by Tsingke Biotechnology.

The primary antibodies included FBW7 antibody (Cat# A301‐720A; Bethyl Laboratories), MCL1 antibody (Cat# SC‐12756; Santa Cruz Biotechnology), MCL1 antibody (Cat# D261457; Sangon), GAPDH antibody (SC‐47724; Santa Cruz Biotechnology), β‐tubulin antibody (Cat# 10068‐1‐AP; Proteintech), HA tag Polyclonal antibody (Cat# 51064‐2‐AP; Proteintech) and DDDDK Tag Rabbit Polyclonal Antibody (Cat# 20543‐1‐AP; Proteintech). Secondary antibodies were Goat peroxidase‐conjugated anti‐Mouse IgG (Cat# 115‐035‐003; Jackson ImmunoResearch) and anti‐Rabbit IgG (Cat# 111‐035‐144; Jackson ImmunoResearch).

Irinotecan (Cat# T6228; Targetmol), SN38 (Cat# T1703; Targetmol), Marinopyrrole A (Maritoclax, Cat# T11944; Targetmol) and MG132 (Cat# MB5137‐1; Meilunbio) were dissolved in dimethylsulfoxide (DMSO); puromycin (Cat# MB2005; Meilunbio) was dissolved in sterile deionized water. These reagents were diluted to appropriate concentrations with cell culture medium for treatment.

### 2.3 Western Blot

Cells and tissue were harvested and lysed by RIPA buffer (Cat# 9806S; Cell Signaling Technology) with phosphatase and protease inhibitor cocktail (Cat# C001/002/003; TargetMol) and incubated on ice for 30 min. Then the lysis was centrifuged at 13000 g, 4°C for 10 min. Quick Start Bradford 1× Dye Reagent (Cat# 5000205; Bio‐Road) was used for total protein quantification and normalization. The lysis supernatants were collected and boiled with 5× SDS loading buffer. Different concentrations of SDS‐PAGE gels were applied to separate proteins according to their molecular weight. All the proteins were then transferred on 0.45 μm (Cat# IPVH00010; Millipore) PVDF membrane and blocked by 5% non‐fat milk for an hour at room temperature to reduce non‐specific binding with antibody. The primary antibodies were prepared according to the recommended dilution and incubated on a shaker at 4°C overnight. Membranes were washed three times by 1× TBST and then incubated with HRP‐conjugated secondary antibody for an hour at room temperature. After washing with 5× TBST three times, immunoreactive proteins on membranes were visualized by Omni‐ECL Pico Light Chemiluminescence Kit (Cat# SQ202; Epizyme Biotech) in ChemiDoc XRS+ System (Bio‐tek). The result was analyzed by Image Lab Software and quantified by Image J Software.

### 2.4 CCK8 Assay

Colorectal cancer cells were seeded in a 96‐well plate. SN38 and Maritoclax were diluted in gradient doses by cell culture medium and treated cells for 48 h. Cell Counting Kit‐8 (CCK8, Cat# MA0218; Meilunbio) was used to detect cell viability. The absorbance of each well was measured spectrophotometrically at a wavelength of 490 nm using the SpectraMAX iD5 Multi‐mode microplate reader (Sunnyvale, CA, USA). The IC50 value was determined by interpolation from the dose–response curves. Results represent the median of three separate experiments and were analyzed by Excel and GraphPad Prism 9.

### 2.5 RNA Extraction, cDNA Synthesis, and Real Time Quantitative Polymerase Chain Reaction (RT‐qPCR)

Total RNA was extracted with the RNAiso Plus (Cat# 9109; Takara) and cDNA was synthesized using the EasyScript One‐Step gDNA Removal and cDNA Synthesis SuperMix (Cat# AE311; TransGen Biotech) according to the manufacturer's protocol. The mRNA expression levels were quantified by RT‐RT‐qPCR using 2× Universal SYBR Green Fast RT‐qPCR Mix (Cat# RK21203; ABclonal) and the mRNA expression levels were determined by the comparative CT (∆∆Ct) method. β‐actin was used as the endogenous control gene, as indicated. The primer sequences used are listed in Table S2. The RT‐RT‐qPCR program included Hot‐start Taq DNA polymerase activation at 95°C (3 min) followed by 40 cycles of DNA denaturation (95°C for 5 s), annealing/extension (60°C for 32 s). All reactions were performed using a QuantStudioTM5 96‐well thermal cycler (Applied Biosystems by Thermo Fisher Scientific).

### 2.6 Patents Information

Paraffin‐embedded tumor slides from patients were collected from the Affiliated Hospital of Qingdao University; the pathologic diagnosis and treatment history of two CRC patients were listed in Table S3. *FBW7* gene wild‐type and mutant CRC patients were identified through whole genome sequencing by Geneseq Biotechnology Co. Ltd. (Nanjing, China); the detailed information of whole genome sequencing was presented in Table S4 and the main gene mutations in FBW7 wild‐type and R465C‐mutant patients were presented in Tables S5 and S6, respectively.

### 2.7 Genomic PCR and Sequencing

Genomic DNA in patient‐derived tumor tissue slides was isolated by using TIANamp FFPE DNA Kit (Cat# DP331; TIANGEN). 1 μL out of 50 μL genomic DNA preparation was amplified by PCR using KOD‐Plus‐Neo (Cat# KOD‐401; Toyobo) and primer pairs FBW7 R465 genomic region: 5′‐CCAGTGTCTGAGAACATTAGTGGGAC‐3′; 5′‐ACAGGAAGCTGACAACACTAGCAAATG‐3′. Sequencing was performed by Tsingke Biotechnology.

### 2.8 Immunohistochemistry

Briefly, the IHC procedure involved baking paraffin sections at 60°C for 2 h, followed by deparaffinization in xylene and hydration through an ethanol series. Antigen retrieval was performed in citrate‐EDTA buffer (95°C–100°C, 20 min), followed by endogenous peroxidase blocking with 3% H_2_O_2_. Sections were blocked with 5% BSA, then incubated with primary antibody (4°C overnight) and HRP‐conjugated secondary antibody (37°C, 30 min). DAB development was monitored microscopically, followed by hematoxylin counterstaining. After dehydration through graded alcohols and xylene clearing, sections were mounted with resin [34]. Images were acquired and analyzed using ImageViewer and ImageJ software, with protein expression quantified as integrated optical density (IOD/Area) from three representative fields per sample.

### 2.9 Gene Knockout, Knockdown and Generation of Stable Cell Lines

The gRNA targeting the FBW7 gene was designed at CHOPCHOP and cloned into lentiCRISPRv2 containing expression cassettes of hSpCas9. The sequences of FBW7 sgRNA are 5′‐CACCGgggcaccagtcgttaacaag‐3′ and 5′‐CACCGccattccacttgttaacgac‐3′. To generate FBW7‐knockout monoclonal cells, colorectal cancer cells were transfected with the LentiCRISPRv2‐sgFBW7 using Lipofectamine 2000. After 24 h incubation, transfected cells were treated with 1 μg/mL puromycin for 48 h and 100 survived cells were seeded into a 96‐well plate. Individual colonies were microscopically confirmed, trypsinized, and expanded. FBW7 knockout efficiency was validated at least three times by Western blot analysis, with wild‐type cells serving as controls.

Lentiviral particles were generated by co‐transfecting HEK293T cells using plasmids (pLVX‐C‐HA ‐FBW7 R465C, pLKO.1‐shFBW7 or lentiCRISPRv2‐sgFBW7) along with packaging plasmids pMD2.G (Cat# 12259; Addgene) and psPAX2 (Cat# 12260; Addgene) at a 4:1:3 mass ratio. The viral supernatant was harvested 48–72 h after transfection, centrifuged at 1288 g for 10 min to remove cell debris, and filtered through 0.45 mm filter (Cat# FF345‐50pcs; Sartorius). To generate FBW7‐knockdown and R465C overexpressed cells, colorectal cancer cells seeded in a 6‐well plate were infected by lentivirus and then cultured in a medium containing 1 μg/mL puromycin for 7 days to kill the uninfected cells. FBW7‐knockdown and R465C‐ovexpressing efficiency was validated at least three times by Western blot analysis, pLVX‐C‐HA, pLKO.1‐Scrambled or lentiCRISPRv2‐sgCtrl (Gao et al., *Proc Natl Acad Sci USA*. 2017;11) served as controls.

### 2.10 AlphaFold Modeling Analysis

The modeling of c‐Myc7 FBW7 wild‐type, and R465C‐mutated protein structures was conducted by AlphaFold (https://alphafoldserver.com/). The structure prediction was performed by the standard AlphaFold pipeline. For further analysis, structures with the best prediction quality were selected and named the best‐lowest model according to a predicted local distance difference test (pLDDT). Preparation of molecular graphics images and minimal distance measurements between atoms of c‐Myc7 FBW7 wild‐type, and R465C‐mutated protein were carried out in PyMol version 2.3.0 (Schrödinger, New York, NY, USA).

### 2.11 Co‐Immunoprecipitation (Co‐IP)

HA‐ and/or Flag‐tagged plasmids were transfected into 293 T cells by using PEI. After transfection for 24–48 h, cells were lysed on ice for 40 min using RIPA buffer. A 10% aliquot of the lysate supernatant was reserved as Input control. The remaining lysates were normalized by protein concentration and volume, then incubated overnight at 4°C with anti‐HA or anti‐Flag antibody. Protein A/G beads were added for 3 h at 4°C, followed by three washes with NETN buffer (100 mM NaCl 20 mM Tris‐Cl (pH 8.0) 0.5 mM EDTA 0.5% (v/v) NP‐40) and boiled with 2× loading buffer at 95°C for 10 min. Western blot was performed to detect the protein–protein interactions.

### 2.12 Animal Experiment

All animal procedures were approved by the Institutional Animal Care and Use Committee (IACUC) and conducted in accordance with its guidelines. Female BALB/c nude mice (5‐week‐old) were obtained from Henan Skobes Biotechnology Co. Ltd. and subcutaneously inoculated with 3 × 10^6^ colorectal cancer cells suspended in 100 μL saline per mouse. Tumor volume was calculated using the formula: (length × width^2^)/2 (mm^3^). Treatment commenced when tumors reached > 100 mm^3^. Drug administration was performed via intraperitoneal injection using a freshly prepared solution containing: 10% DMSO/drugs +40% PEG300 + 5% Tween‐80 + 45% saline, vortexed before use. Irinotecan (35 mg/kg) and/or Maritoclax (5 mg/kg) were administered every 4 days. Tumor volumes and body weights were recorded every other day. Mice were sacrificed when tumor volume > 1500 mm^3^ or diameter > 20 mm; tumor tissues were excised for photographic documentation and then stored at −80°C or fixed in 4% paraformaldehyde (Cat# BL539A; Biosharp).

### 2.13 Statistical Analysis

All experiments were repeated with at least three biological replicates, data expressed as the mean ± SD. Differences between two data sets were analyzed by two‐way ANOVA or Student's *t*‐test with *p* < 0.05 considered statistically significant.

## 3 Results

### 3.1 Loss or Low Expression of FBW7 Promotes SN38 Resistance in CRC Cells

To investigate the impact of FBW7 expression level on SN38 resistance in CRC cells, we established FBW7‐knockdown RKO cells, FBW7‐knockout HCT‐116 and DLD1 cells using CRISPR/Cas9 gene editing, as well as FBW7‐knockdown HCT‐116 cells through RNA interference. CCK8 assays showed that both the loss or low expression of FBW7 significantly decreased CRC cells' sensitivity to SN38 compared with the control groups.

**FIGURE 1:**
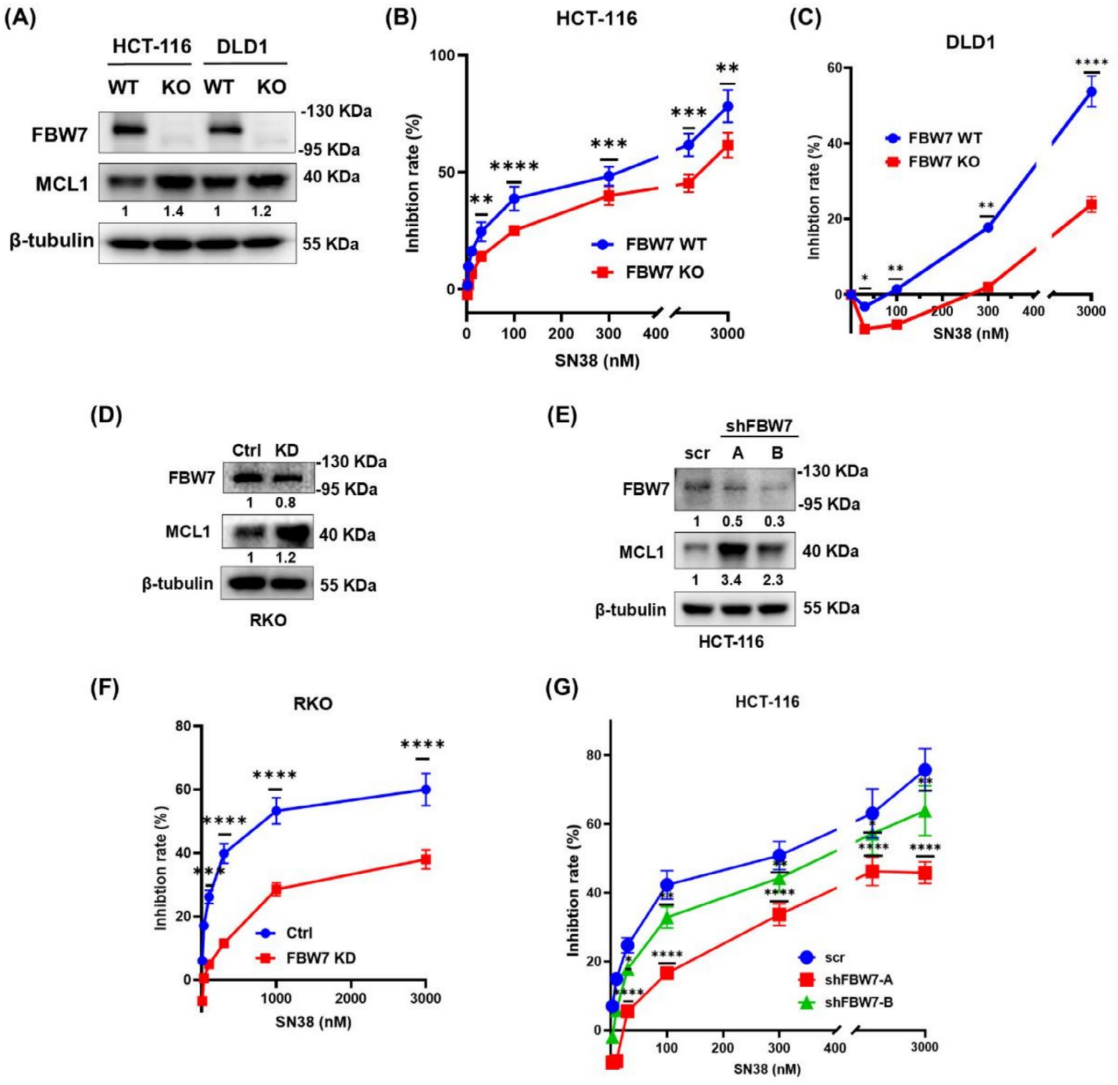
Loss or low expression of FBW7 promoted SN38 resistance in CRC cells. (A) The protein levels of FBW7 and MCL1 in FBW7 wild‐type (WT) and knockout (KO) HCT‐116 and DLD1 cells were detected by Western blot. CCK8 assay was performed to measure absorbance and calculate the inhibition ratio after wild‐type (WT) and FBW7 ‐ knockout (KO) (B) HCT‐116 and (C) DLD1 cells were treated by increasing doses of SN38 for 48 h. The protein levels of FBW7 and MCL1 in (D) control (Ctrl) and FBW7‐knockdown (KD) RKO and (E) scramble control (scr) and FBW7‐knockdown (shFBW7) HCT‐116 cells were detected by Western blot. CCK8 assay was performed to measure absorbance and calculate the inhibition ratio after (F) control (Ctrl) and FBW7‐knockdown (KD) RKO and (G) scramble control (scr) and FBW7‐knockdown (shFBW7) HCT‐116 cells were treated by increasing doses of SN38 for 48 h. Data are expressed as the mean ± SD (*n* = 3, representing three independent experiments). *p* values were calculated by using Student's *t*‐test; ****p < 0.0001, ***p < 0.001, **p < 0.01, *p < 0.05.

In detail, FBW7‐knockout and wild‐type HCT‐116 and DLD1 cells (Figure 1A) were treated with gradient concentrations of SN38 (0–3000 nM) for 48 h. Our results showed that the half‐maximal inhibitory concentration (IC50) of FBW7‐knockout HCT‐116 cells (1.4 μM) was approximately 2‐fold higher than that of wild‐type cells (0.77 μM) (Figure 1B), while the IC50 of DLD1 FBW7 ‐ knockout cells (48.96 μM) was about 24‐fold higher than that of wild‐type cells (2.18 μM) (Figure 1C).

We then measured the inhibition ratios of the FBW7‐knockdown group and control group cells (Figure 1D,E) after treatment with gradient concentrations of SN38 (0–3000 nM) for 48 h by CCK8 assays. The results showed that in RKO cells, the IC50 value of the FBW7‐knockdown group (14.47 μM) was approximately 16‐fold higher than that of the control group (0.90 μM) (Figure 1F). In HCT‐116 cells, the IC50 values of the FBW7‐knockdown group (1.90/0.48 μM) were approximately 1.6 to 6.6‐fold higher than that of the control group (0.29 μM) (Figure 1G).

These results confirmed that loss or low expression of FBW7 promotes SN38 resistance in CRC cells. Notably, MCL1 protein levels were consistently upregulated in both FBW7‐knockout and FBW7‐knockdown CRC cells (Figure 1A,D,E).

### 3.2 FBW7 Mutation Conferred Resistance to SN38 in CRC Cells

**FIGURE 2:**
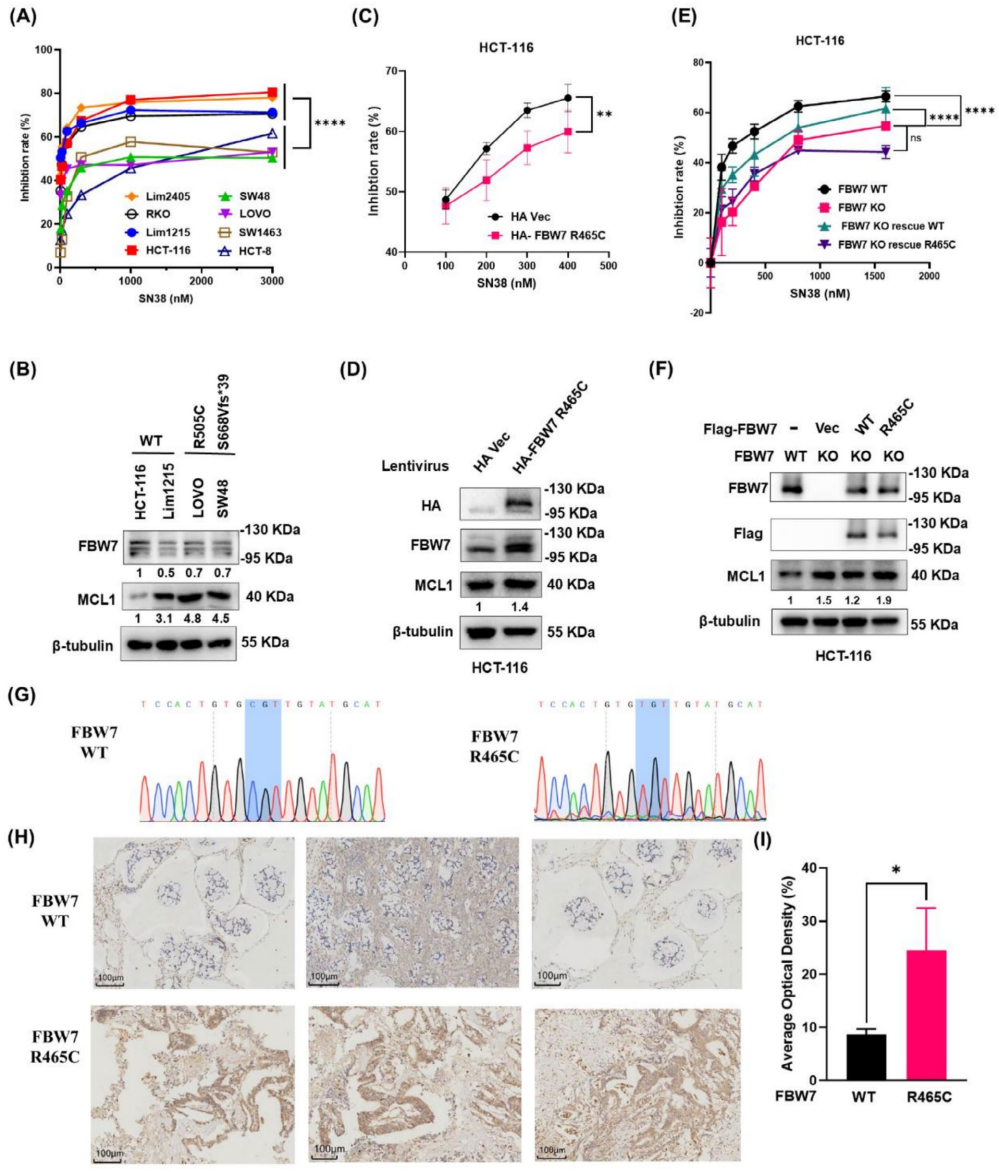
FBW7 mutation conferred resistance to SN38 in CRC cells. (A) The inhibition ratios of SN38 on FBW7 wild‐type (Lim1215, HCT‐116, Lim2405 and RKO) and mutant (LOVO, SW1463, SW48 and HCT‐8) CRC cells were detected by CCK8 assay after treatment with SN38 for 48 h. (B) The expression levels of FBW7 and MCL1 proteins in FBW7 wild‐type (Lim1215 and HCT‐116) and mutant (LOVO and SW48) CRC cells were detected by Western blot. (C) The inhibition ratio of SN38 treatment for 48 h on HA‐Vec and HA‐FBW7 R465C‐overexpressed HCT‐116 cells was detected by CCK8 assay. (D) The expression of FBW7 and MCL1 protein levels in HA‐Vec and HA‐FBW7 R465C‐overexpressed HCT‐116 cells were detected by Western blot. (E) The inhibition ratio of SN38 treatment for 48 h on FBW7 wild‐type (WT), knockout (KO), KO rescued Flag‐FBW7 wild‐type and KO rescued Flag‐FBW7 R465C HCT‐116 cells was detected by CCK8 assay. (F) The expression of FBW7 and MCL1 protein levels in cells from (E) was detected by Western blot. (G) DNA sequencing of the targeted genomic regions in CRC patient‐derived tumor tissue slides highlighting the WT and corresponding FBW7 R465C‐mutant sequences. (H) The protein levels of MCL1 in FBW7 wild‐type (WT) and R465C mutant tumor samples were detected by immunohistochemistry. (I) Three pictures of each sample were collected for quantification. Average Optical Density (%) = Integrated optical density/Area. Data are expressed as the mean ± SD (*n* = 3, representing three independent experiments). *p* values were calculated by using Student's *t*‐test or Two‐way ANOVA; ns: *P* > 0.05, ****p < 0.0001, **p < 0.01, *p < 0.05.

To elucidate the correlation between FBW7 mutation and SN38 sensitivity, we determined the IC50 of SN38 in various CRC cell lines with wild‐type and mutant FBW7 by CCK8 assays. The results demonstrated that in FBW7 wild‐type cell lines (Lim1215, HCT‐116, Lim2405, and RKO), the IC50 values of SN‐38 were 8.77, 25.03, 62.20, and 87.72 nM, respectively. In contrast, FBW7‐mutant cell lines (LOVO, SW1463, SW48, and HCT‐8) exhibited significantly higher IC50 values (634.70, 868.70, 1195.6, and 1883.09 nM, respectively; *p* < 0.0001, Figure 2A), suggesting that FBW7 mutations significantly reduce SN38 sensitivity in CRC cells. Notably, the R505C, S668Vfs*39, and R479Q mutations are all located within the WD40 domain of the FBW7 protein, which is responsible for substrate recognition and binding [16]. Western blot analysis revealed significantly elevated MCL1 protein expression in FBW7‐mutant (LOVO/SW48) versus FBW7‐wild‐type (HCT‐116/Lim1215) cell lines (Figure 2B).

Furthermore, we examined the association between the high‐frequency R465 mutation in FBW7 and irinotecan resistance. FBW7 wild‐type and R465C‐mutant tumor samples were collected from CRC patients; the clinical records showed that the patient harboring the FBW7 R465C mutation exhibited disease progression after irinotecan‐based chemotherapy (Table S3), suggesting this mutation may also induce irinotecan resistance.

To validate the FBW7 R465C‐induced chemoresistance, we established HCT‐116 cells stably expressing HA‐tagged FBW7 R465C through lentiviral infection. CCK8 assays (0–400 nM, 48 h) revealed a significantly diminished SN38 response in R465C‐overexpressed cells (*p* < 0.01, Figure 2C). The MCL1 protein levels showed a 1.4‐fold increase in R465C‐overexpressed cells compared to vector control cells (Figure 2D). Since FBW7 has been reported to form heterodimers in vivo [35], we wonder whether R465C‐mutated FBW7 affects the expression level or function of the wild‐type FBW7. Our results revealed that overexpression of the FBW7 R465C failed to cause obvious downregulation of wild‐type FBW7 in HCT‐116 cells (Figure S1A–C). Intriguingly, co‐expression of FBW7 R465C with wild‐type FBW7 resulted in a marked accumulation of MCL1 compared with the expression of wild‐type FBW7 alone (Figure S1D–F). In line with our findings, the FBW7 R465C has been reported to exert a dominant‐negative effect that abrogates the function of its wild‐type counterpart [36]. Together, our results indicate that R465C overexpression impairs the function of wild‐type FBW7 without altering its expression level.

We also rescued wild‐type or R465C‐mutant FBW7 in FBW7‐knockout HCT‐116 cells, CCK8 assays found that the inhibition ratios in FBW7‐knockout cells were significantly lower than that in wild‐type counterpart (*p* < 0.0001, Figure 2E), Rescuing R465C‐mutant FBW7 showed no significant changes compared to FBW7‐KO cells (Figure 2E). Similarly, the MCL1 level increased by 50% in FBW7‐KO HCT‐116 cells compared to the wild‐type cells (Figure 2F). In contrast to wild‐type FBW7, the R465C‐mutated FBW7 failed to restore MCL1 depletion in FBW7‐knockout cells (Figure 2F). Consistently, we found the MCL1 expression was also elevated in the patient's tumor sample harboring the FBW7 R465C mutation compared to the wild‐type counterpart (*p* < 0.05; Figure 2G–I).

### 3.3 FBW7 R465C Mutation Weakened Its Binding to MCL1

**FIGURE 3:**
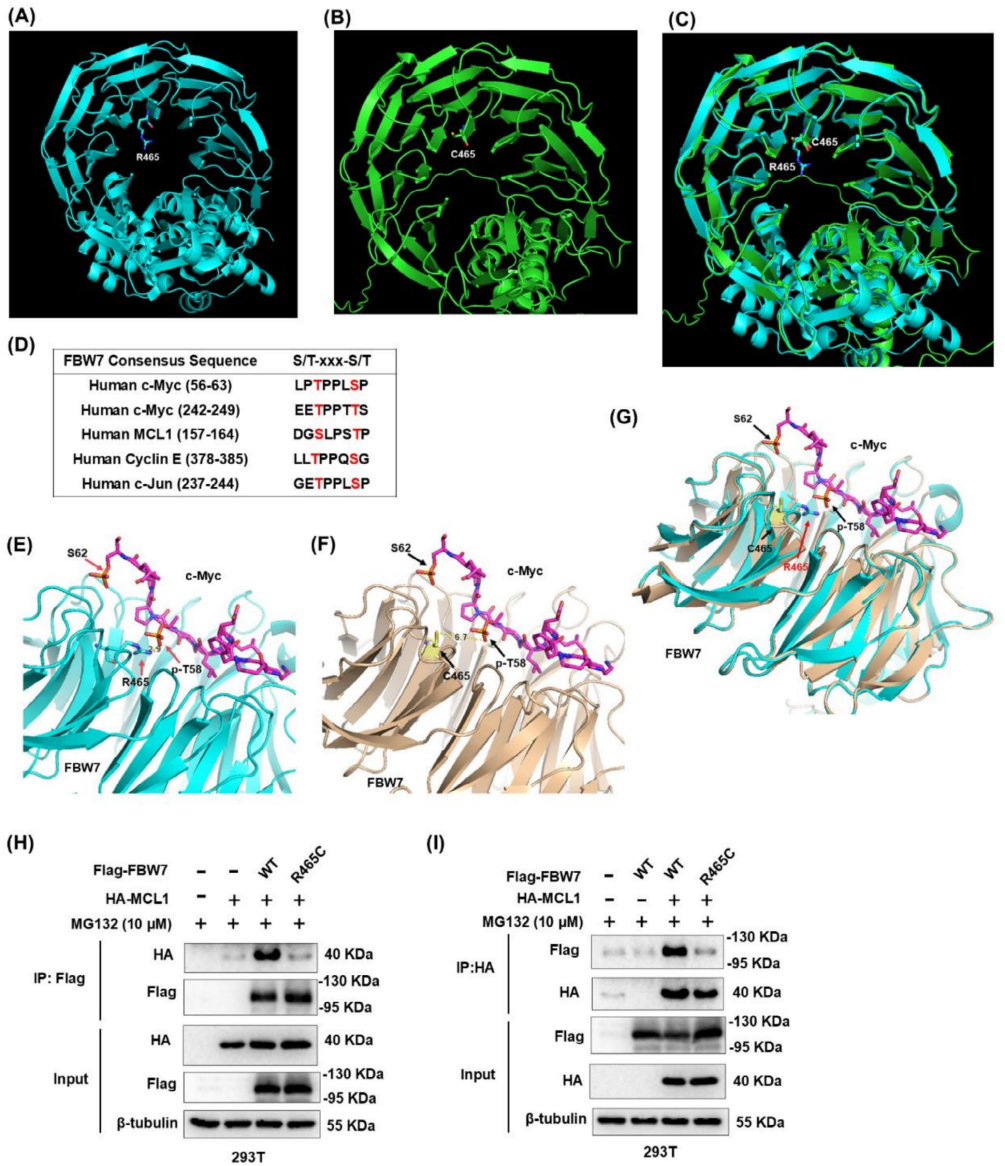
FBW7 R465C mutation weakened its binding to MCL1. The three‐dimensional structures were predicted using AlphaFold3 of FBW7 R465 (A, wild‐type, blue), FBW7 C465 (B, mutated, green), and their merged form (C). (D) FBW7 recognized consensus sequence (S/T‐xxx‐S/T) among its substrates including c‐Myc, MCL1, c‐Jun, and Cyclin E. Close‐up view of the interface formed between FBW7 and the c‐Myc T58 phospho‐degron (PDB: 7t1z) simulated by AlphaFold: (E) FBW7 R465 (wild‐type) and c‐Myc (residues 56 to 63); (F) FBW7 C465 (mutated) and c‐Myc (residues 56 to 63); (G) merged form of (E, F). FBW7 is colored in blue(wild‐type) or khaki (C465, mutated) with the c‐Myc‐interacting sequence shown in purple. The c‐Myc diphosphorylated degron p‐T58 and p‐S62 residue are colored in orange. Hydrogen bonds and electrostatic interactions are indicated by yellow dashed lines. (H, I) Co‐IP was performed to detect the binding of FBW7 wild‐type (WT) or R465C with MCL1.

To investigate the functional change of R465C‐mutated FBW7, we used AlphaFold (https://alphafold.ebi.ac.uk/) to analyze the conformational change in the FBW7 WD40 domain after R465 mutated to cysteine. However, there is no obvious structural change between R465 (wild‐type) and C465 (mutated) (Figure 3A–C).

FBW7 recognizes and binds to its substrates only after their phosphorylation at conserved phospho‐degron motifs, known as Cdc4 phospho‐degrons (CPDs) [14]. Intriguingly, FBW7 CPDs are a consensus sequence (S/T‐xxx‐S/T) among its substrates including c‐Myc, MCL1, c‐Jun, and Cyclin E (Figure 3D) [37, 38]. Due to the lack of a co‐crystal structure of the FBW7 and MCL1 complex, we introduced the R465C mutation into FBW7 based on the FBW7‐c‐Myc complex structure (PDB: 7t1z) [38]. It has been reported that S62 is phosphorylated priming T58; this diphosphorylation regulates c‐Myc protein stability, and c‐Myc p‐T58 makes hydrogen bonds and electrostatic contacts with the R465 residue of FBW7 [39]. Our results revealed that the c‐Myc p‐T58 degron is anchored to the top surface of the FBW7 WD40 domain; the crystal structure of wild‐type FBW7 (R465) in complex with the phosphorylated degron peptide (residues 56 to 63) was at 2.9 Å resolution (Figure 3E), while the C465‐mutated FBW7 was at 6.7 Å resolution (Figure 3F). The R‐to‐C mutation caused the amino acid side chain to change and rotate away from c‐Myc p‐T58, which is supposed to weaken their direct interaction (Figure 3G). We therefore propose that the FBW7 R465C mutation may similarly reduce the binding affinity with MCL1's FBW7 phospho‐degron (residues 157 to 164) at p‐S159, leading to impaired MCL1 recognition and binding.

To verify the binding affinity of R465C‐mutated FBW7 and MCL1, we performed Co‐immunoprecipitation (Co‐IP) and found that the R465C mutation severely impaired the FBW7 interaction with MCL1, whereas wild‐type FBW7 retained robust interaction (Figure 3H,I). These findings suggested that the FBW7 R465C mutation potentially mediates SN38 resistance through MCL1 upregulation.

### 3.4 SN38 Regulates MCL1 Expression in CRC Cells

**FIGURE 4:**
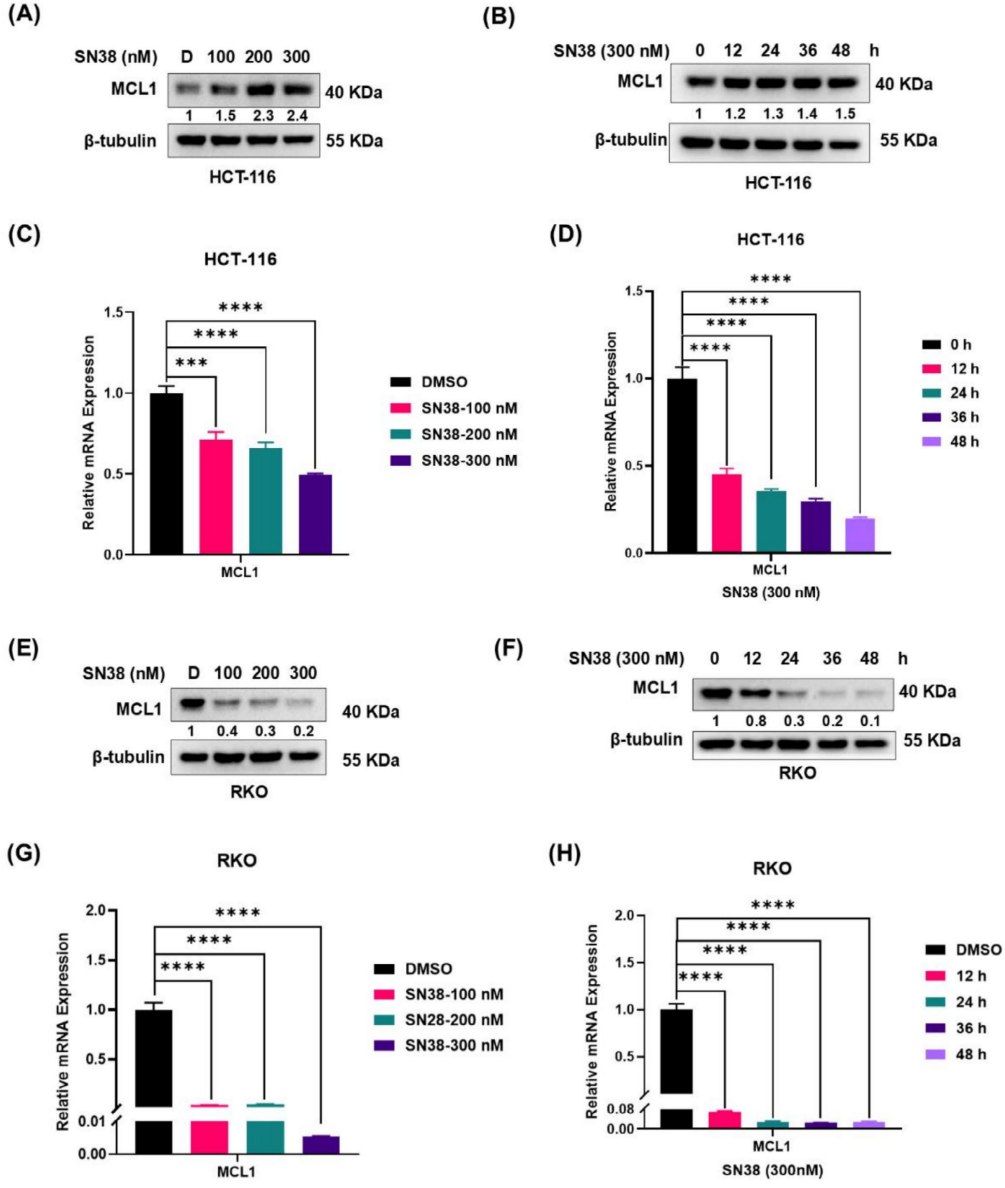
SN38 regulates MCL1 expression in CRC cells. The protein levels of MCL1 in HCT‐116 cells (A) treated with 100/200/300 nM SN38 for 48 h or (B) treated with 300 nM SN38 for 0/12/24/36/48 h were detected by Western blot. The mRNA levels of MCL1 in HCT‐116 cells (C) treated with 100/200/300 nM SN38 for 48 h or (D) treated with 300 nM SN38 for 0/12/24/36/48 h were detected by RT‐qPCR. The protein levels of MCL1 in RKO cells (E) treated with 100/200/300 nM SN38 for 48 h or (F) treated with 300 nM SN38 for 0/12/24/36/48 h were detected by Western blot. The mRNA levels of MCL1 in RKO cells (G) treated with 100/200/300 nM SN38 for 48 h or (H) treated with 300 nM SN38 for 0/12/24/36/48 h were detected by RT‐qPCR. “D” represents DMSO (vehicle control). Data are expressed as the mean ± SD (*n* = 3, representing three independent experiments). *p* values were calculated by using Student's *t*‐test, ****p < 0.0001, ***p < 0.001.

To establish whether MCL1 is a key effector of SN38's antitumor mechanism, then validate its targeting as a strategy to overcome chemoresistance, we analyzed MCL1 protein and mRNA levels using Western blot and qPCR, respectively, following SN38 treatment (Figure 4).

Our results demonstrated that SN38 treatment differentially regulates MCL1 expression in CRC cell lines. In HCT‐116 cells, SN38 increased MCL1 protein levels in a dose‐ and time‐dependent manner (Figure 4A,B), despite downregulating MCL1 mRNA (Figure 4C,D). Differently, RKO cells showed decreased MCL1 protein (Figure 4E,F) and mRNA levels (Figure 4G,H) following SN38 treatment. These findings suggest cell type‐specific regulation of MCL1 by SN38, involving both transcriptional and post‐transcriptional mechanisms.

### 3.5 MCL1 Overexpression Resulted in SN38 Resistance

**FIGURE 5:**
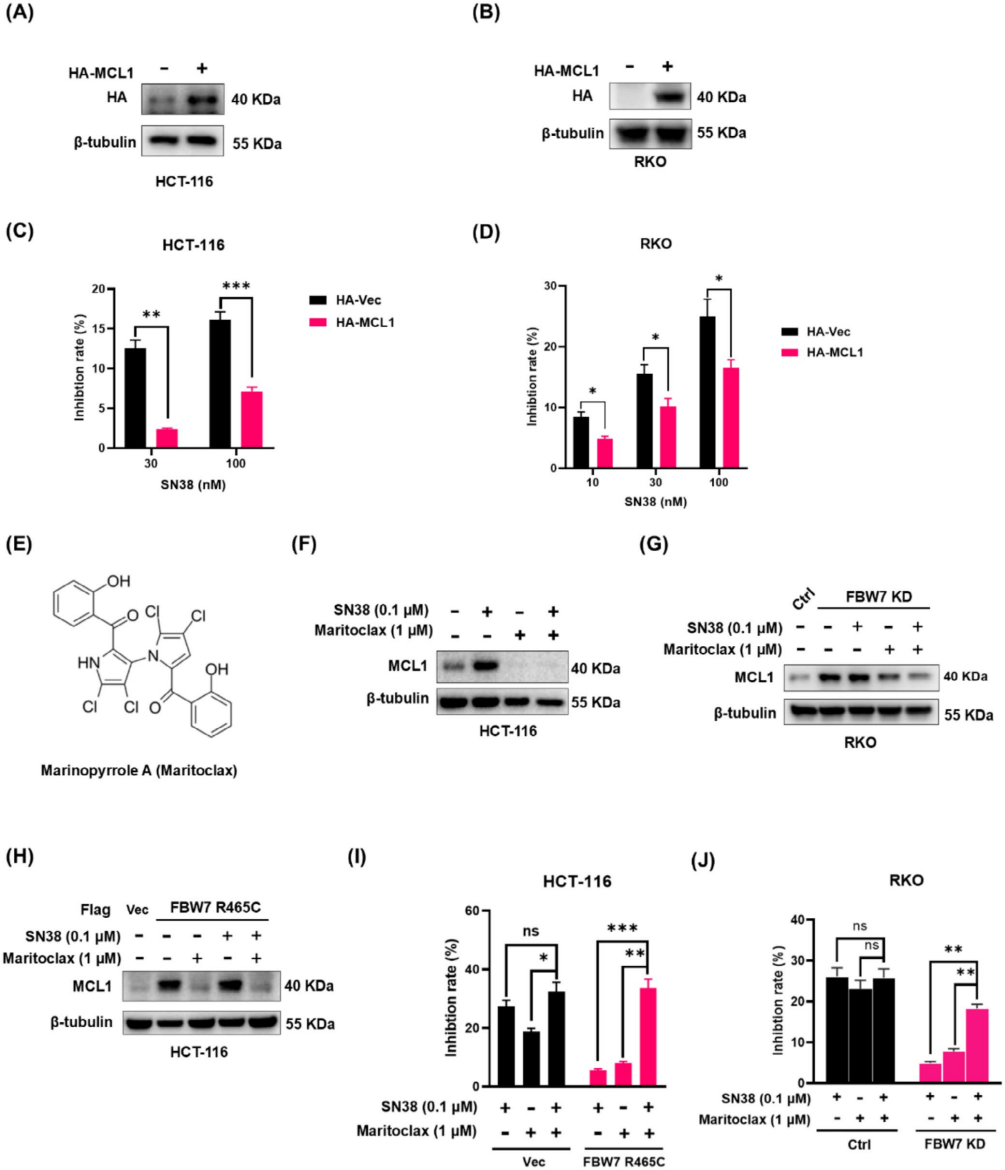
MCL1 overexpression resulted in SN38 resistance. Western blot was performed to detect the expression of HA‐MCL1 plasmid in (A) HCT‐116 cells and (B) RKO cells after transfection for 16 h. CCK8 assay was performed to detect the absorbance and calculate the inhibition ratio of 30/100 nM SN38 treatment for 48 h on (C) HCT‐116 cells and (D) RKO cells transfected with HA‐Vec or HA‐MCL1 plasmids. (E) The chemical structure of Marinopyrrole A (Maritoclax). Western blot was performed to detect the protein levels of MCL1 in (F) HCT‐116 cells, (G) FBW7 knockdown and control RKO cells, (H) Flag‐Vec‐ or Flag‐FBW7 R465C‐overexpressed HCT‐116 cells treated with 0.1 μM SN38 and/or 1 μM Maritoclax for 48 h. CCK8 assay was performed to detect the absorbance and calculate the inhibition ratio of 0.1 μM SN38 and/or 1 μM Maritoclax treatment for 48 h on (I) Flag‐Vec‐ or Flag‐FBW7 R465C‐overexpressed HCT‐116 cells, (J) FBW7 knockdown and control RKO cells. Data are expressed as the mean ± SD (*n* = 3, representing three independent experiments). *p* values were calculated by using Student's *t*‐test, ns: *P* > 0.05, ***p < 0.001, **p < 0.01, *p < 0.05.

To determine whether MCL1 upregulation mediates SN38 resistance in CRC cells, we overexpressed HA‐tagged MCL1 in HCT‐116 and RKO cells (Figure 5A,B) and measured drug sensitivity by CCK8 assay (Figure 5C,D). The results revealed that MCL1 overexpression significantly decreased cell sensitivity to SN38 compared to control cells. At 30 nM and 100 nM SN38 treatments, the inhibition rates in MCL1‐overexpressed HCT‐116 groups were 2.35% and 7.13%, significantly lower than those in control groups (12.53% and 16.09%, *p* < 0.01, Figure 5C), respectively. Similarly, at 10, 30 and 100 nM SN38 treatment, the inhibition rates in MCL1‐overexpressed RKO groups were 4.87%, 10.18% and 16.47%, significantly lower than those in control groups (8.50%, 15.55% and 24.97%, *p* < 0.05, Figure 5D). This functional validation through MCL1 overexpression confirmed its protective role, establishing MCL1 as a resistance factor.

We then targeted this resistance mechanism by combining Maritoclax (Figure 5E), a selective MCL1 inhibitor that disrupts MCL1 binding to the Bim BH3 α‐helix (IC50 = 10.1 μM) and induces MCL1 protein degradation through the ubiquitin‐proteasome pathway [29]. Our results showed that Maritoclax treatment effectively eliminated the SN38 (Figure 5F), FBW7 ‐ knockdown (Figure 5G)‐, FBW7 R465C overexpression (Figure 5H) ‐induced elevated MCL1 levels in CRC cells.

Moreover, the combination of SN38 (0.1 μM) and Maritoclax (1 μM) significantly enhanced the inhibition in FBW7 R465C‐overexpressed HCT‐116 cells and FBW7 ‐ knockdown RKO cells compared to either treatment alone (*p* < 0.01, Figure 5I,J), while no significant synergistic effect was observed in control cells (*p* > 0.05, Figure 5I,J). In control HCT‐116 cells, the inhibition rates of SN38 alone, Maritoclax alone and their combination were 27.38%, 18.72% and 32.46%, respectively (Figure 5I). In contrast, FBW7 R465C‐overexpressed cells exhibited a 6‐fold greater response to the combination treatment compared to SN38 alone, with inhibition rates of 5.56%, 7.97%, and 33.58%, respectively. In control RKO cells, the inhibition rates of SN38 alone, Maritoclax alone and their combination were 26.17%, 23.08% and 25.92%, respectively, while FBW7 knockdown RKO cells exhibited a 3.7‐fold greater response to the combination treatment compared to SN38 alone, with inhibition rates of 4.93%, 7.85% and 18.27%, respectively (Figure 5J).

These findings provide strong experimental evidence for combining irinotecan/SN38 with Maritoclax in FBW7‐deficient CRC.

### 3.6 Maritoclax Enhanced the Antitumor Effect of Irinotecan on FBW7 R465C‐Overexpressed CRC In Vivo

**FIGURE 6:**
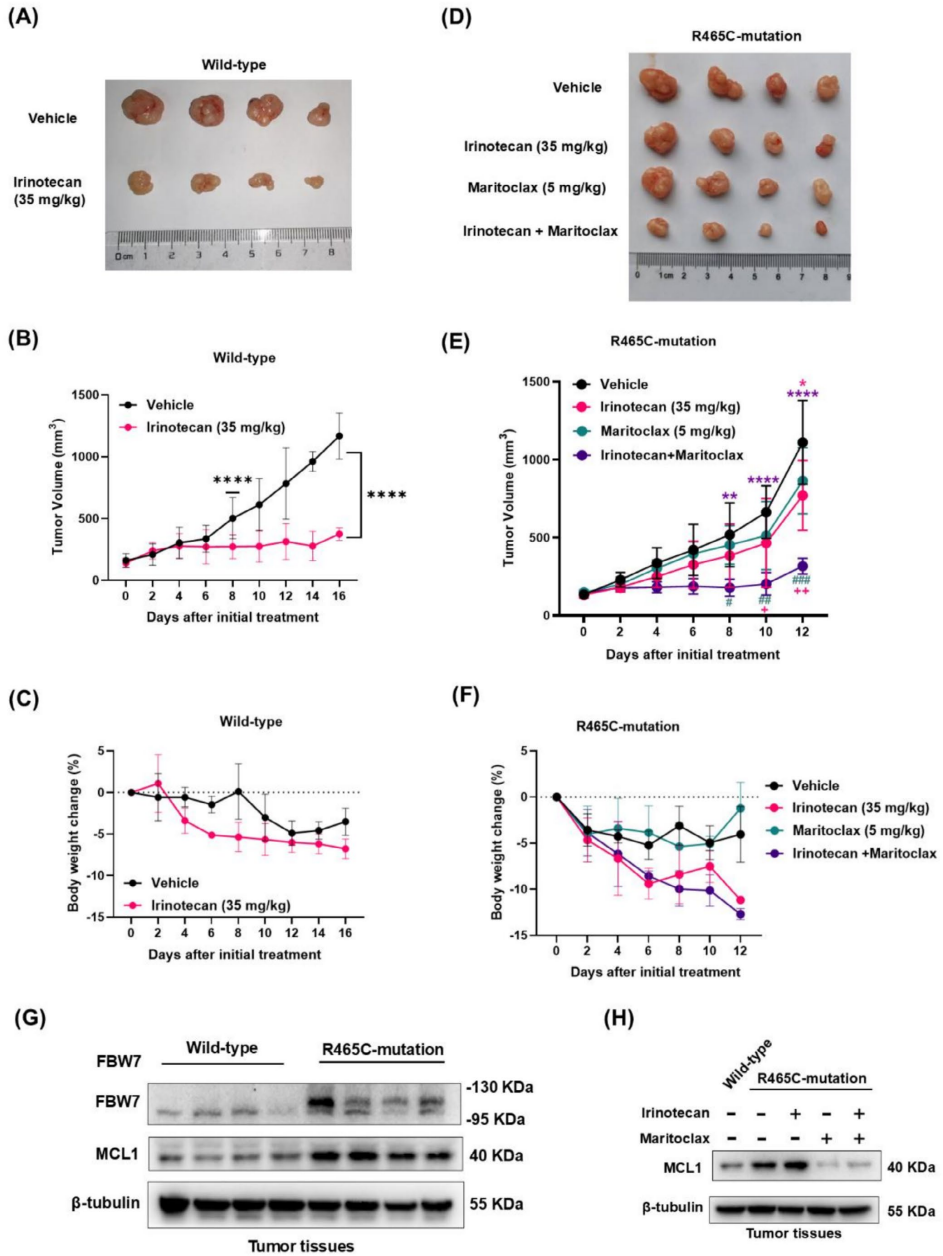
Maritoclax enhanced the antitumor effect of irinotecan on FBW7 R465C‐overexpressed CRC in vivo. HA‐Vec (wild‐type group) or HA‐FBW7 R465C ‐overexpressed (R465C‐mutation group) HCT‐116 cells were subcutaneously injected into 5‐week‐old female BALB/c nude mice. Drugs were administered every 4 days after tumor volume reached 100 mm^3^. Nude mice in the wild‐type group were assigned to vehicle (DMSO) or irinotecan (35 mg/kg) treatment groups and those in R465C‐mutation group were divided into four groups that treated with vehicle (DMSO), irinotecan (35 mg/kg), Maritoclax (5 mg/kg), or irinotecan (35 mg/kg) plus Maritoclax (5 mg/kg), respectively. Nude mice were sacrificed when tumor volume reached 1500 mm^3^ or diameter reached 20 mm. (A, D) Tumors were dissected and recorded by photographed. (B, E) Tumor volumes (length*width^2^/2) and (C, F) body weights of mice were recorded every other day during treatment. Western blot was performed to detect the protein level of MCL1 in tumor tissues from (G) the wild‐type and R465C‐mutation control groups; (H) the wild‐type control group and R465C‐mutation groups including control, irinotecan monotherapy, Maritoclax monotherapy, and combination therapy. Data are expressed as the mean ± SD (*n* = 3, representing three independent experiments). *p* values were calculated by using Two‐way ANOVA or Student's *t*‐test, the coloration of the *p*‐value markers is consistent with the color scheme of each corresponding group. ns: *p* > 0.05, ****p < 0.0001, ***p < 0.001, **p < 0.01, *p < 0.05 vs. the vehicle group; ###*p* < 0.001, ##*p* < 0.01, #*p* < 0.05 vs. the irinotecan group; ++*p* < 0.01, +*p* < 0.05 vs. the Maritoclax group.

Since loss, low expression, and mutation of FBW7 all result in its functional deficiency and upregulation of MCL1, we established FBW7 R465C‐overexpressed HCT‐116 xenograft models to assess the in vivo therapeutic efficacy of Maritoclax and irinotecan treatment. According to the literature reports, Maritoclax at 20 mg/kg administered every other day significantly inhibits tumor growth [30]. During dose optimization, we found that 30 mg/kg irinotecan combined with 10 mg/kg Maritoclax caused acute death in nude mice, whereas 35 mg/kg irinotecan combined with 5 mg/kg Maritoclax did not induce significant body weight changes (≤ 12%, Figure 6F), and therefore the latter combination was selected for subsequent experiments.

In wild‐type FBW7 xenograft models, irinotecan monotherapy significantly inhibited tumor growth by Day 8 post‐treatment (*p* < 0.0001, Figure 6A,B), without inducing significant body weight loss (< 10%, Figure 6C). In contrast, in the FBW7 R465C mutant xenograft model, irinotecan exhibited significant antitumor effects only by day 12 (*p* < 0.05), whereas Maritoclax monotherapy showed no significant efficacy (Figure 6D,E). Notably, the Maritoclax–irinotecan combination group demonstrated a pronounced reduction in tumor volume on Days 8, 10, and 12 (*p* < 0.01, *p* < 0.0001, respectively; Figure 6E). Compared to irinotecan monotherapy, the combination therapy led to significantly smaller tumors on Days 10 and 12 (*p* < 0.01, *p* < 0.001, respectively). Similarly, when compared to Maritoclax monotherapy, the combination group showed a significant decrease in tumor volume at all measured time points (*p* < 0.05, *p* < 0.01, *p* < 0.001, respectively). Overall, the combination group exhibited superior tumor suppression relative to the vehicle control, irinotecan monotherapy, and Maritoclax monotherapy groups, with a marked reduction in tumor volume (Figure 6D).

We then detected the MCL1 protein levels in tumor tissue by Western blot. Our results showed that the expression of MCL1 was higher in FBW7 R465C‐mutation groups than in the wild‐type (Figure 6G), Maritoclax treatment potently depleted the upregulation of MCL1 (Figure 6H), which corresponded to the results in vitro (Figure 5G,H). These results demonstrate that Maritoclax effectively enhanced the antitumor efficacy of irinotecan in CRC with FBW7 R465C overexpression.

## 4 Discussion

FBW7 serves as a tumor suppressor in CRC, it has been reported that FBW7 mutations [40, 41] and low expression [42, 43, 44, 45] are strongly positively associated with poor prognosis in CRC patients. Previous studies have also demonstrated that the loss of function of FBW7 confers various chemoresistance to CRC, including regorafenib [20], trametinib [21], paclitaxel [22], 5‐fluorouracil (5‐FU) and oxaliplatin [23]. Our data confirm that FBW7 deficiency also promotes irinotecan resistance, highlighting its crucial role in treatment response.

Notably, we observed disease progression in a CRC patient carrying the FBW7 R465C mutation following irinotecan therapy. Our experiments demonstrated that FBW7 R465C significantly attenuates irinotecan/SN38 efficacy in both cellular and animal models; restoration of FBW7 R465C in FBW7‐knockout CRC cells fails to rescue SN38 sensitivity. Similarly, Tong et al. showed that compared to wild‐type cells, FBW7‐knockout cells exhibited significantly enhanced resistance to regorafenib treatment. Also, they showed the tumor‐derived mutants of FBW7 (R465C, R479Q, and R505C) failed to restore regorafenib sensitivity in FBW7‐knockout cells [20].

Furthermore, mutations resulting in the amino acid substitutions R465C, R479Q, and R505C are predicted to alter both hydrophobic and electrostatic surface interactions of FBW7, which are likely to affect substrate binding [46]. Our Co‐IP assays demonstrated markedly reduced FBW7 R465C‐MCL1 binding compared to wild‐type FBW7, leading to impaired degradation and consequent upregulation of MCL1 in R465C‐mutated patient‐derived CRC tumor tissues and experimental models, which is consistent with the previous finding [15, 20]. Although the FBW7‐MCL1 co‐crystal structure awaits future studies, the FBW7 R505C, R479Q, S668Vfs*39 and R465C‐induced MCL1 upregulation underscores the essential role of these amino acid residues in mediating FBW7‐MCL1 interactions. Comparably, we exhibited reduced binding affinity of R465C‐mutated FBW7 with c‐Myc p‐T58 degron, whose sequence is a consensus among FBW7 substrates, such as c‐Jun, Cyclin E and MCL1. Previous structural analysis has uncovered that Cyclin E p‐T380 degron, c‐Myc p‐T58 degron and p‐T244 degron make a network of hydrogen bonds and electrostatic contacts with R465, R479 and R505 on FBW7; the three degrons align perfectly with the corresponding region, highlighting the critical roles of these arginine residues in substrate recognition and binding from a structural perspective [38].

Intriguingly, we ectopically overexpressed the FBW7 R465C in HCT‐116 cells and observed a consequent increase in MCL1 protein levels, Overexpression of FBW7 R465C did not significantly suppress the expression of wild‐type FBW7 but rather interfered with its function, suggesting a dominant‐negative effect between wild‐type and R465C‐mutated FBW7 on MCL1 upregulation. The dominant‐negative mechanism may be attributed to the in vivo interaction among different FBW7 isoforms via their N‐terminal D‐domain motifs, which normally enhance the ubiquitylation activity of the SKP1‐CUL1‐F‐box (SCF) complex [35]. Since most primary tumors harbor only a single FBW7 mutation while retaining a wild‐type allele, they exhibit a state of haploinsufficiency in tumor suppression [47]. If FBW7 operates predominantly as a homodimer or heterodimer in vivo, a single mutant allele could be sufficient to disable its function. However, whether physiological expression levels of the R465C mutation are sufficient to exert this dominant‐negative effect on wild‐type FBW7 remains to be investigated.

As a critical FBW7 substrate, MCL1 overexpression drives therapeutic resistance [48, 49]. Our findings demonstrated that MCL1 upregulation promotes irinotecan/SN38 resistance, while its inhibition by the selective MCL1 inhibitor Maritoclax effectively degrades FBW7 deficiency‐induced MCL1 accumulation and restores irinotecan/SN38 sensitivity both in vitro and in vivo. Maritoclax represents a novel class of MCL1 inhibitor that likely binds MCL1's p4 hydrophobic pocket in a Noxa‐mimetic fashion, competitively displacing Bim and triggering MCL1 degradation [29]. While our and most reported studies focus on its MCL1‐inhibitory effects [29, 30, 31, 33], emerging evidence suggests Maritoclax may also exert biological activity through MCL1‐independent mechanisms [32]. For example, it enhances TRAIL‐mediated apoptosis by upregulating DR5 through CHOP induction while suppressing c ‐ FLIP via miR‐708; these effects have been consistently demonstrated in multiple cancer cell lines (renal, lung, and hepatocellular carcinomas) [50]. Further exploration of these novel mechanisms is warranted, highlighting Maritoclax's promising potential for clinical therapy.

## 5 Conclusions

In summary, our findings demonstrate that Maritoclax, a selective MCL1 inhibitor, effectively overcomes FBW7 deficiency‐driven irinotecan resistance in CRC by targeting and degrading aberrantly accumulated MCL1. These results suggest Maritoclax as a promising combination therapy to improve treatment outcomes for CRC patients with FBW7 dysfunction, addressing a critical unmet need in this high‐risk population.

## Author Contributions

**Qian Lin:** conceptualization (equal), data curation (equal), formal analysis (equal), investigation (equal), project administration (equal), supervision (equal), validation (equal), visualization (equal), writing – original draft (equal). **Shuting Liu:** investigation (equal), validation (equal), visualization (equal), writing – original draft (equal). **Hongfei Jiang:** funding acquisition (equal), investigation (equal), resources (equal). **Shasha Wang:** funding acquisition (equal), resources (equal). **Yixin Duan:** conceptualization (equal), data curation (equal), funding acquisition (equal), project administration (equal), resources (equal), supervision (equal), visualization (equal), writing – review and editing (equal).

## Funding

This research was funded by the National Natural Science Foundation of China, grant number 82372806; Natural Science Foundation of Shandong Province, China, grant number ZR2024QH181; the Postdoctoral Fellowship Program (Grade C) of China Postdoctoral Science Foundation, grant number GZC20240770; the National Natural Science Foundation of China, grant number 82202848; Wu Jieping Medical Foundation, China, grant number 320.6750.2024‐18.66.

## Ethics Statement

The study was conducted in accordance with the Declaration of Helsinki, and approved by the Ethics Committee of the Affiliated Hospital of Qingdao University (protocol code QYFYEC2024‐198, August 28, 2024) for studies involving humans. The animal study protocol was approved by the Ethics Committee of Medical College of Qingdao University (protocol code QDU‐AEC‐2022105, February 28, 2022) for studies involving animals.

## Consent

Patient consent was waived due to the retrospective study design (using anonymized or untraceable data/biospecimens that cannot be linked to personal identity).

## Conflicts of Interest

The authors declare no conflicts of interest.

## Supporting information


**Table S1:** Genotypes of different CRC cell lines.
**Table S2:** Primer sequences in RT‐qPCR.
**Table S3:** Pathologic diagnosis and treatment history of two CRC patients.
**Table S4:** Detailed information of Whole genome sequencing.
**Table S5:** Information of the main gene mutations in the FBW7 wild‐type patient.
**Table S6:** Information of the main gene mutations in the FBW7 R465C‐mutant patient.

**Figure S1:** Overexpression of FBW7 R465C did not significantly suppress the expression of wild‐type FBW7 but rather interfered with its function. (A) Briefly, plasmids expressing HA‐FBW7 R465C or vector control was transfected into HCT‐116 cells. Cells were lysed by RIPA buffer and divided into two parts: 50% cell lysis were retained as “Cell lysis Before IP HA”; excessive HA antibody and Protein A/G beads was added into other 50% cell lysis to IP HA‐tagged FBW7 T465C, the 50% supernatant lysis after centrifuged were retained as “Cell lysis After IP HA”. (B, C) Co‐Immunoprecipitation and Western blot were performed to detect the expression of HA‐FBW7 R465C, FBW7 and MCL1 in HCT‐116 cells before and after IP HA. Red arrow refers to the basic wild‐type FBW7 in HCT‐116 cells. (D) Western blot was performed to detect the protein expression in FBW7‐knockout HCT116 cells co‐transfected with plasmids that express HA‐MCL1, wild‐type FBW7 (WT) and increasing amounts of FBW7 R465C.

## Data Availability

The data that support the findings of this study are available from the corresponding author upon reasonable request.
