# Supplementary material for: Maritoclax Overcomes FBW7 Deficiency‐Driven Irinotecan Resistance in Colorectal Cancer by Targeting MCL1
Source: Cancer Med. 2025 Nov 28;14(23):e71419. doi: 10.1002/cam4.71419 (PMC12661461; doi:10.1002/cam4.71419)
Supplement: Supplementary file 1 — Table S1: Genotypes of different CRC cell lines. Table S2: Primer sequences in RT‐qPCR. Table S3: Pathologic diagnosis and treatment history of two CRC patients. Table S4: Detailed information of Whole genome sequencing. Table S5: Information of the main gene mutations in the FBW7 wild‐type patient. Table S6: Information of the main gene mutations in the FBW7 R465C‐mutant patient. [file CAM4-14-e71419-s001.docx]

**Table S1. Genotypes of different CRC cell lines.**

| **CRC Cell lines** | **ApC** | **Tp53** | **KRAS** | **pIK3CA** | **FBW7** |
| --- | --- | --- | --- | --- | --- |
| HCT-116 | WT | WT | p.G13D | p.H1047R | WT |
| Lim1215 | WT | WT | WT | WT | WT |
| RKO | WT | WT | WT | p.H1047R | WT |
| Lim2405 | p.A697V  p.V2194Ffs*5 | WT | WT | WT | WT |
| DLD1 | p.R727M  p.K993N  p.I1417Lfs*2  p.R2166* | WT | p.G13D | p.E545K  p.D549N  p.R741= | WT |
| LOVO | p.R1114*  p.T1430fs  p.R2816Q | WT | p.G13D | WT | R505C |
| SW48 | p.R2714C | WT | WT | p.G914R | p.S668Vfs*39 p.L152= |
| HCT-8 | WT | WT | p.G13D | p.E545K  p.D549N | c.501+29048 T>A |
| SW1463 | p.V452Sfs*7 | p.R248Q | p.G12C | WT | p.R479Q |
|  | | | | | |

WT: wild-type; *: nonsense mutation; fs: frame shift; =: coding silent; >: substitution.

**Table S2. Primer sequences in RT-qPCR.**

| **Primer** | **Sequences (5’→3’)** |
| --- | --- |
| MCL1-F | ccaagaaagctgcatcgaaccat |
| MCL1-R | cagcacattcctgatgccacct |
| β-actin-F | caccattggcaatgagcggttc |
| β-actin-R | aggtctttgcggatgtccacgt |

**Table S3. Pathologic diagnosis and treatment history of two CRC patients.**

| **FBW7 genotype** | **Gender** | **Age** | **Pathologic diagnosis** | **Treatment history** |
| --- | --- | --- | --- | --- |
| Wide-type | Female | 36 | Rectal carcinoma,  Pelvic and peritoneal metastases | 2015.02-2015.08: Oxaliplatin+Fluorouracil+Cetuximab (Erbitux), no Progression.  2016.05-2016.11: surgery, Oxaliplatin+Fluorouracil+Cetuximab（Erbitux).  2017-2018: drug discontinuation, relapse and progression. Pelvic and peritoneal metastases |
| R465C-mutant | Male | 47 | Stage IV  sigmoid colorectal adenocarcinoma,  liver metastases | 2018.03.21: XELOX, no Progression  2018.04.14-2018.07.17: mFOLFOX6+Cetuximab(Erbitux), Progressive disease.  **2018.08.06-2018.08.23: FOLFIRI +Avastin(Bevacizumab), Progressive disease.**  2018.10.15: surgery. |

**Table S4. Detailed information of Whole genome sequencing.**

| **Sample Type** | **Blood precipitation，plasma，FFPE rectal tissue** |
| --- | --- |
| Detection range | All exons of 425 genes and, part of its related intron regions, almost 1.28Mb site of alternative splicing regions and specific microsatellite regions. All kinds of mutation (site mutation, deletion-Insertion mutation, copy number variation and fusion mutation) were detected in these regions, genotype of mismatch repair, MS analysis and tumor mutation burden information were also provided. |
| Detection platform | Illumina Hiseq |
| Method | Next-generation sequencing |
| Reference Genome | GRCh37/hg19 |

**Table S5. Information of the main gene mutations in the FBW7 wild-type patient.**

| **Gene name** | **Variation** | **Mutation Type** | **Plasma Abundance** | **Tissue Abundance** |
| --- | --- | --- | --- | --- |
| *ANKRD62* | ANKRD62  ~ZNF516&ZNF3 6 fusion | ANKRD62: exon6  ~ZNF516&ZNF236 | - | 2.8% |
| *BRCA2* | p.S2976N  Exon 22 mutation | p.S2976N (c.G8927A) | 4.6% | 12.7% |
| *CCND1* | Gene amplification | - | - | 4.0 folds |
| *FGF19* | Gene amplification | - | 1.9 folds | 3.6 folds |
| *FGFR1* | Gene amplification | - | - | 2.3 folds |
| *GRM8* | p.A155E  Exon 1 mutation | p.A155E (c.C464A) | 3.0% | 9.4% |
| *KRAS* | Gene amplification | - | - | 3.9 folds |
| *Tp53* | c.G993+1A  Intron 9 mutation | c.G993+1A | 8.6% | 35.2% |
| *ZNF703* | Gene amplification | - | - | 2.5 folds |

**Table S6. Information of the main gene mutations in the FBW7 R465C-mutant patient.**

| **Gene name** | **Variation** | **Mutation Type** | **Plasma Abundance** | **Tissue Abundance** |
| --- | --- | --- | --- | --- |
| *ApC* | p.W423X  Exon 10 truncating mutation | p.W423X (c.G1268A) | 58.2% | 33.2% |
| *BRAF* | p.N581S  Exon 15 mutation | p.N581S (c.A1742G) | 41.5% | 22.2% |
| *CDKN2A* | p.R131C  Exon 2 mutation | p.R131C (c.C391T) | 38.7% | 25% |
| *DpYD* | p.K34T  Exon 2 mutation | p.K34T (c.A101C) | 38.1% | 23.6% |
| *FBXW7* | p.R465C  Exon 9 mutation | p.R465C (c.C1393T) | 38.9% | 22.3% |
| *FLT1* | p.R1120T  Exon 25 mutation | p.R1120T (c.G3359C) | - | 1.5% |
| *KRAS* | p.L19F  Exon 2 mutation | p.L19F (c.G57T) | 41.9% | 25.8% |
| *SMAD4* | p.G386D  Exon 10 mutation | p.G386D (c.G1157A) | 73.8% | 47.0% |
| *Tp53* | p.H214fs  Exon 6 frameshift mutation | p.H214fs  (c.642_643delTA) | 60.1% | 37.4% |
| *Tp53* | Single-copy deletion | - | Single-copy deletion | - |
