# Supplementary material for: Maritoclax Overcomes FBW7 Deficiency‐Driven Irinotecan Resistance in Colorectal Cancer by Targeting MCL1
Source: Cancer Med. 2025 Nov 28;14(23):e71419. doi: 10.1002/cam4.71419 (PMC12661461; doi:10.1002/cam4.71419)
Supplement: Supplementary file 2 — Figure S1: Overexpression of FBW7 R465C did not significantly suppress the expression of wild‐type FBW7 but rather interfered with its function. (A) Briefly, plasmids expressing HA‐FBW7 R465C or vector control was transfected into HCT‐116 cells. Cells were lysed by RIPA buffer and divided into two parts: 50% cell lysis were retained as “Cell lysis Before IP HA”; excessive HA antibody and Protein A/G beads was added into other 50% cell lysis to IP HA‐tagged FBW7 T465C, the 50% supernatant lysis after centrifuged were retained as “Cell lysis After IP HA”. (B, C) Co‐Immunoprecipitation and Western blot were performed to detect the expression of HA‐FBW7 R465C, FBW7 and MCL1 in HCT‐116 cells before and after IP HA. Red arrow refers to the basic wild‐type FBW7 in HCT‐116 cells. (D) Western blot was performed to detect the protein expression in FBW7‐knockout HCT116 cells co‐transfected with plasmids that express HA‐MCL1, wild‐type FBW7 (WT) and increasing amounts of FBW7 R465C. [file CAM4-14-e71419-s002.docx]

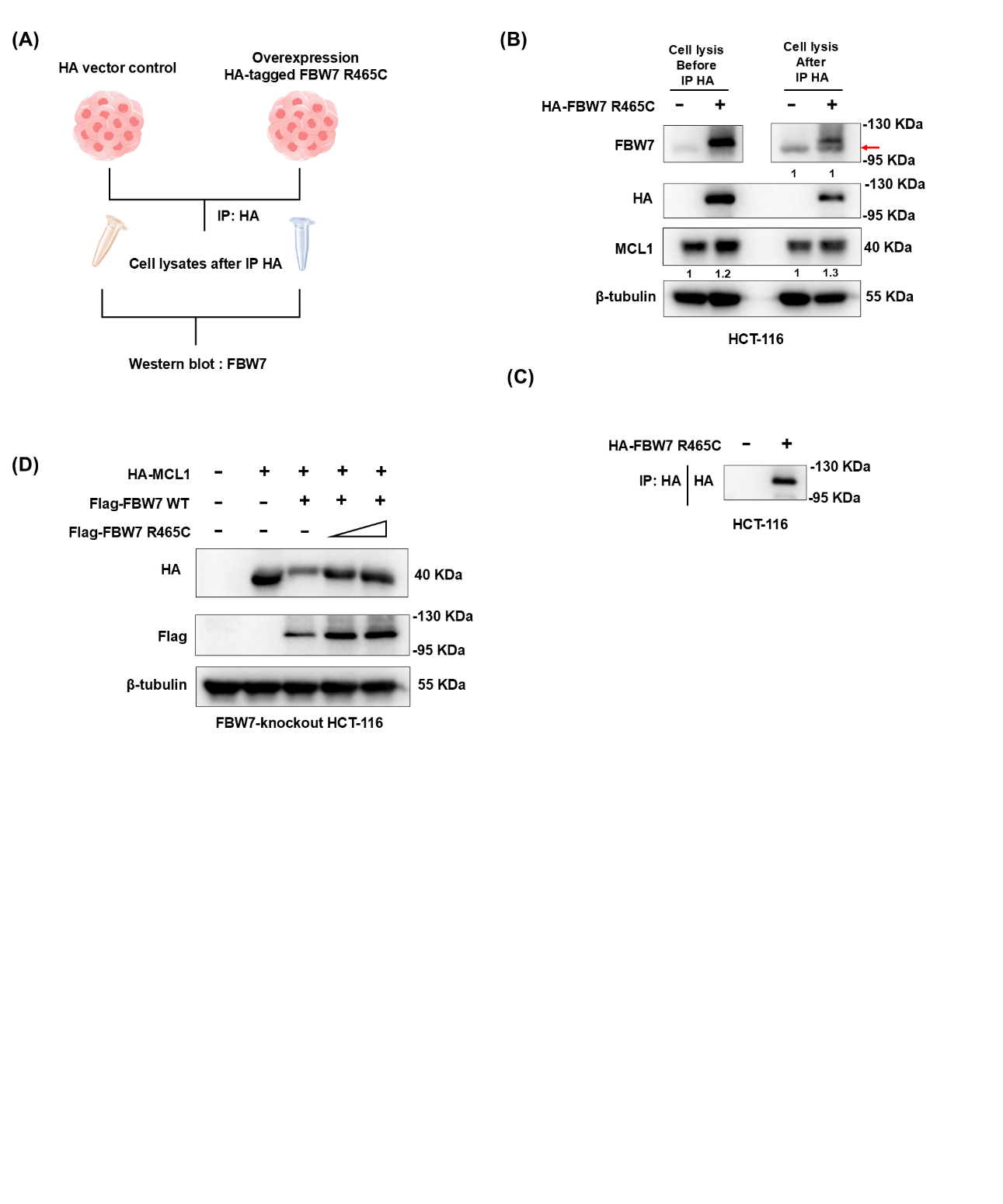


**Figure S1.** **Overexpression of FBW7 R465C did not significantly suppress the expression of wild-type FBW7 but rather interfered with its function. (A)** Briefly, plasmids expressing HA-FBW7 R465C or vector control was transfected into HCT-116 cells. Cells were lysed by RIPA buffer and divided into two parts: 50 % cell lysis were retained as “Cell lysis Before IP HA”; excessive HA antibody and Protein A/G beads was added into other 50 % cell lysis to IP HA-tagged FBW7 T465C, the 50 % supernatant lysis after centrifuged were retained as “Cell lysis After IP HA”. **(B) (C)** Co-Immunoprecipitation and Western blot were performed to detect the expression of HA-FBW7 R465C, FBW7 and MCL1 in HCT-116 cells before and after IP HA. Red arrow refers to the basic wild-type FBW7 in HCT-116 cells. **(D)** Western blot was performed to detect the protein expression in FBW7-knockout HCT116 cells co-transfected with plasmids that express HA-MCL1, wild-type FBW7 (WT) and increasing amounts of FBW7 R465C.
